# Supplementary material for: Half-metre sea-level fluctuations on centennial timescales from mid-Holocene corals of Southeast Asia
Source: Nat Commun. 2017 Feb 10;8:14387. doi: 10.1038/ncomms14387 (PMC5309900; doi:10.1038/ncomms14387)
Supplement: Supplementary Information — Supplementary Figures, Supplementary Tables, Supplementary Note and Supplementary References [file ncomms14387-s1.pdf]

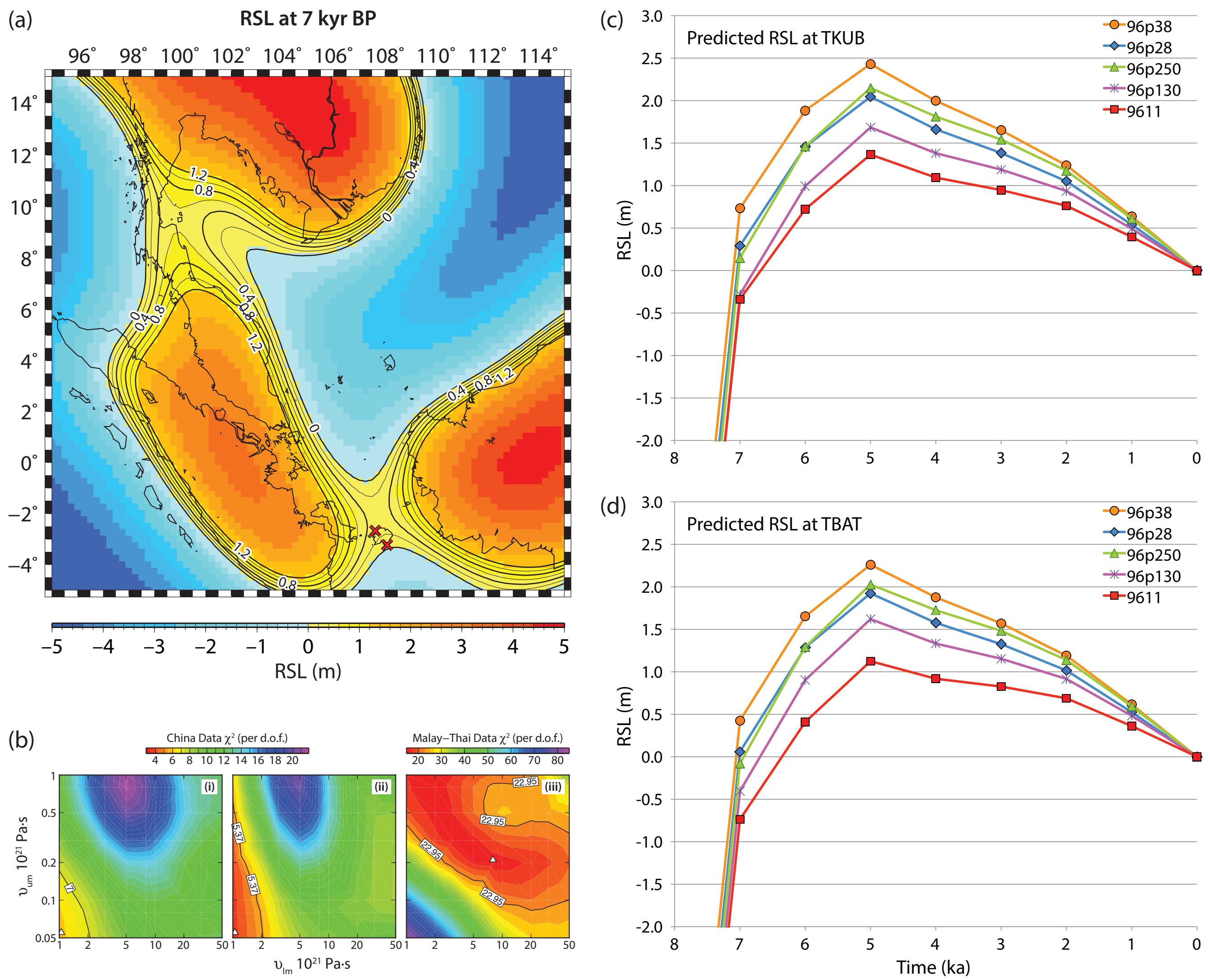

Supplementary Figure 1 | Predicted mid-Holocene changes in RSL due to GIA.
(**a**) Map of the spatially variable RSL at 7 kyr BP predicted by the "96p28" earth model and the ice melting history of Bradley et al.[1]. The locations of the TKUB (northwestern) and TBAT (southeastern) sites are each indicated by an "×". Map created in GMT. (**b**) χ^2 analysis of data–model misfits for RSL predictions using a suite of earth models, from Bradley et al.[1]. Contour plots of χ^2 as a function of upper and lower mantle viscosity (υ_{um} and υ_{lm}, respectively) for a lithosphere thickness of 96 km are shown for (**i**) their complete China data set, (**ii**) a reduced China data set[2], and (**iii**) a data set from the Malay–Thai Peninsula[3]. The black line represents the χ^2 value below which the quality of fit is equivalent at 95% confidence, and the white triangle marks the location of the minimum χ^2 value. Several potential models are listed in Supplementary Table 5. (**c**, **d**) Predicted RSL at the (**c**) TKUB (northwestern Belitung) site and (**d**) TBAT (southeastern Belitung) site for a range of earth models that fit within the 95% confidence limit from the χ^2 analysis for the Malay–Thai Peninsula.

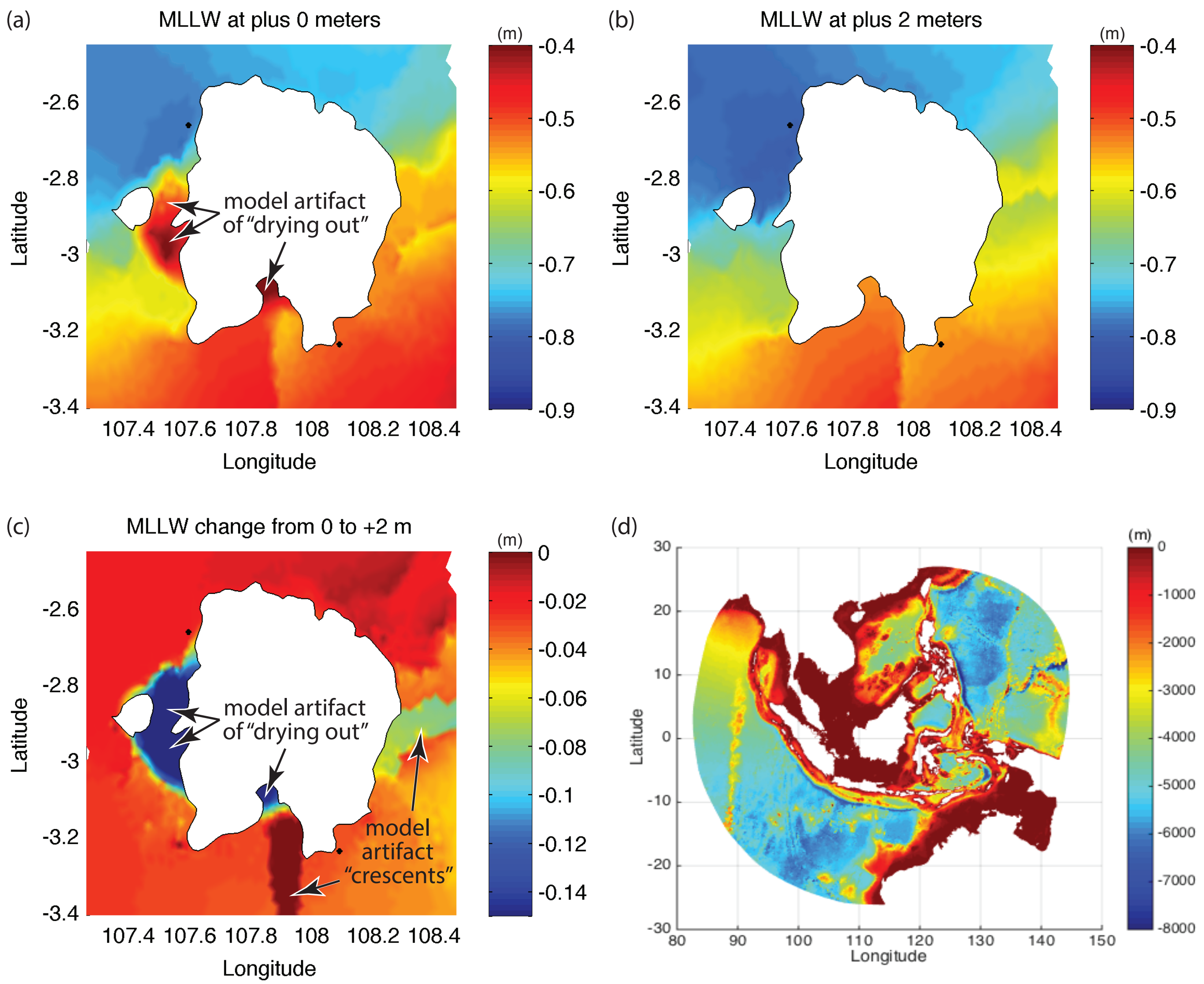

Supplementary Figure 2 | Predicted mid-Holocene changes in tidal range. Model predictions of mean lower low water (MLLW) (**a**) at present and (**b**) under conditions of 2 m higher RSL, as is predicted by GIA for the Belitung region ~7 kyr BP and as is suggested by our data. (**c**) Change in MLLW from the map in (**a**) to the map in (**b**). A negative value in (**c**) indicates that MLLW was lower, and suggests the tidal range was greater, under conditions of slightly higher RSL than present. Artifacts are discussed in the Methods. (**d**) A map of the grid bathymetry used for the modeling. Maps created in MATLAB.

High-Viscosity Rheology

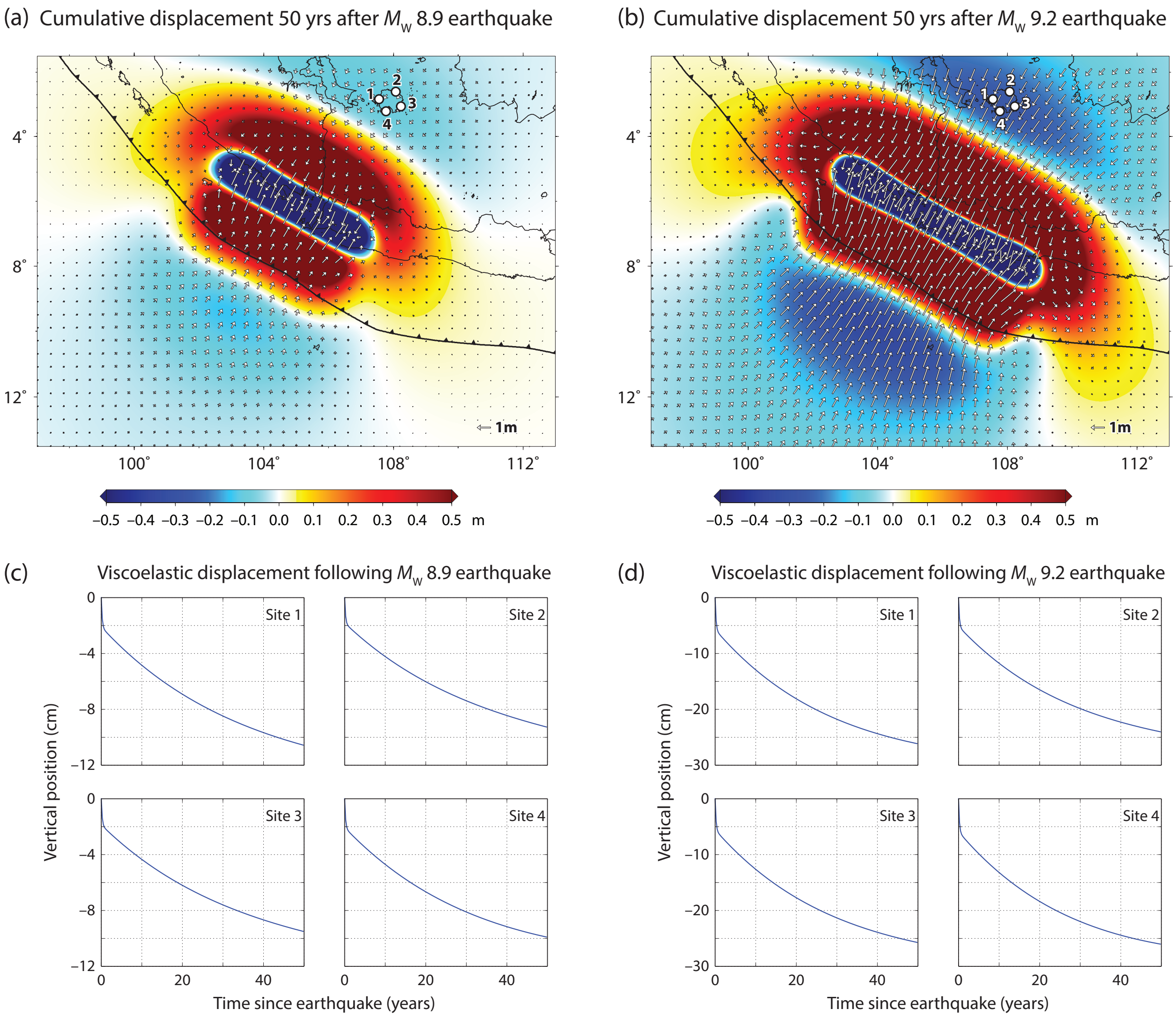

Supplementary Figure 3 | Predicted viscoelastic response to hypothetical megathrust rupture. Legend on next page.

Low-Viscosity Rheology

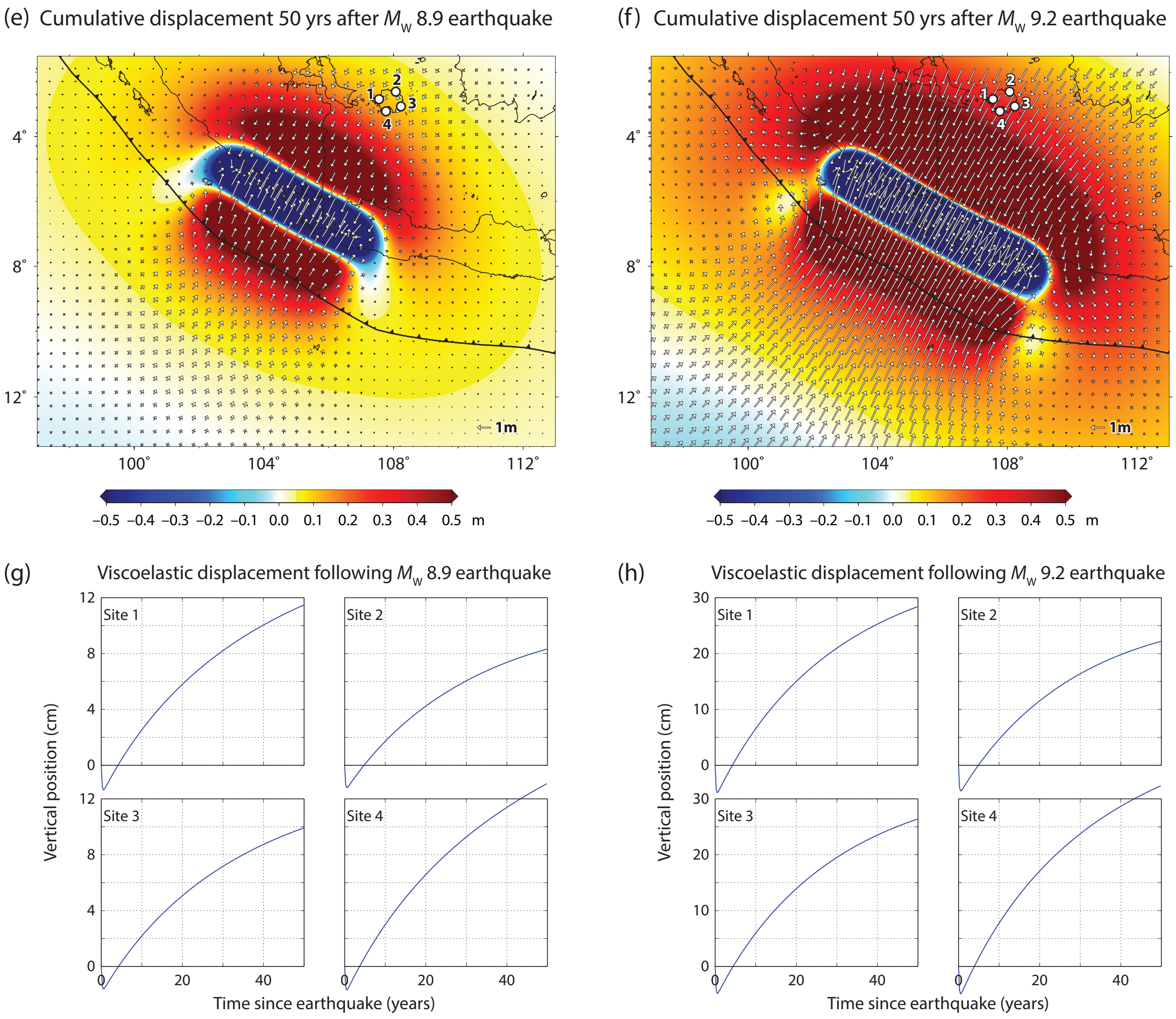

Supplementary Figure 3 | Predicted viscoelastic response to hypothetical megathrust rupture. Predicted cumulative viscoelastic deformation using the higher-viscosity rheology of Pollitz et al.[4,5] (**a–d**) and the lower-viscosity rheology of Panet et al.[6] (**e–h**), 50 yr after (**a**, **e**) a M_W 8.9 rupture and (**b**, **f**) a M_W 9.2 rupture along the portion of the Sunda–Java megathrust closest to Belitung. Arrows on (**a**, **b**, **e**, **f**) represent horizontal displacement (see 1 m scale bar on figure) and color represents vertical displacement. Also shown are time series for four sites on Belitung showing the evolution of predicted vertical deformation following (**c**, **g**) the M_W 8.9 and (**d**, **h**) the M_W 9.2 rupture. The sites are located by number on the maps in (**a**, **b**, **e**, **f**). See Methods for details of the construction of this figure. Maps created in MATLAB.

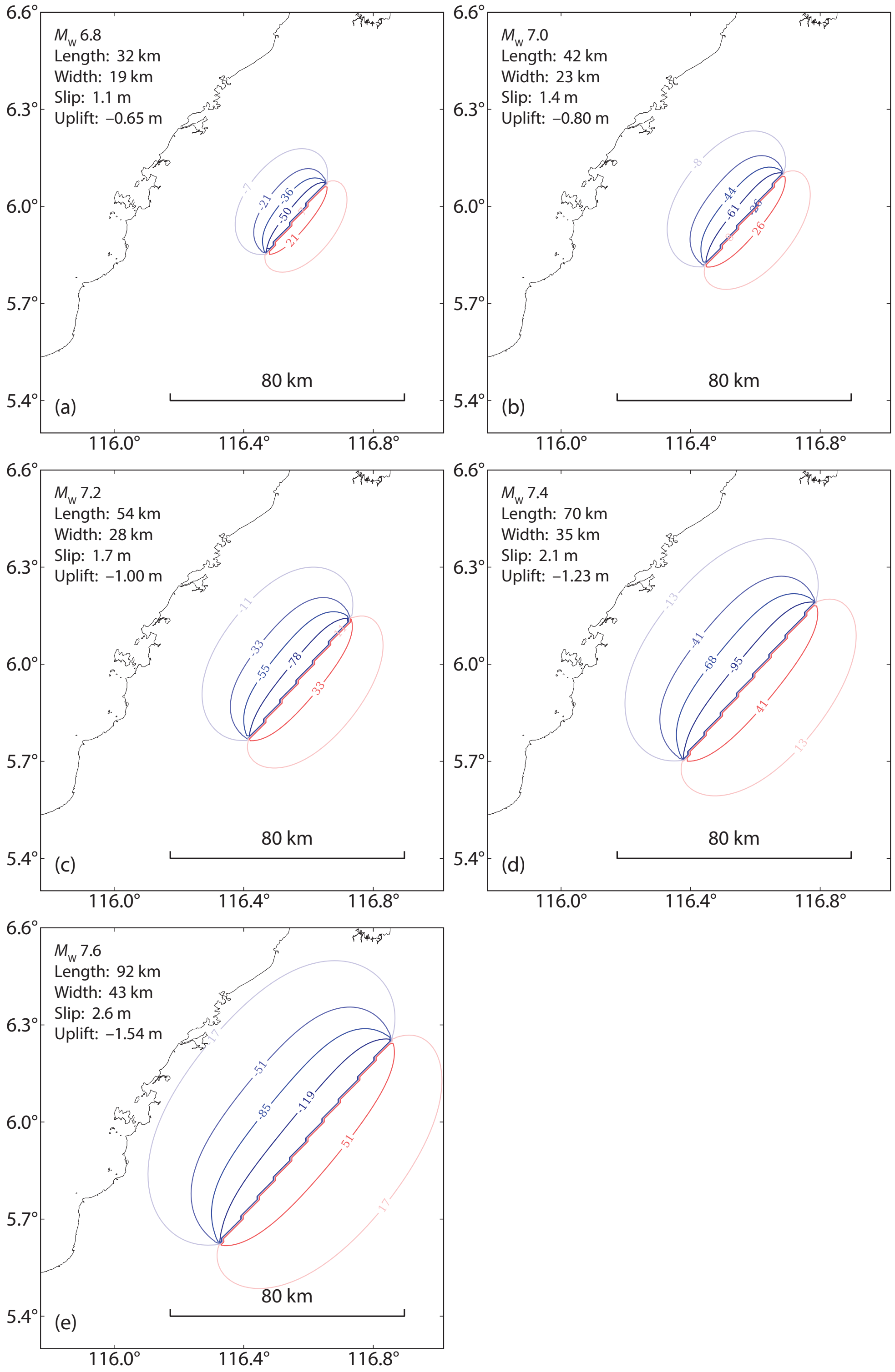

Supplementary Figure 4 | Predicted deformation due to a hypothetical upper-plate fault. Elastic dislocation model predictions of vertical deformation (contour labels in cm) associated with pure dip slip along a fault plane dipping 70° to the northwest. Parameters noted in each panel: moment magnitude; rupture length; rupture width; uniform slip; and peak vertical displacement (either up or down). Only a rupture of $M_W \approx 7.6$ or greater can produce vertical displacement of ~0.6 m or more at sites 80 km apart from one another. Maps created in MATLAB.

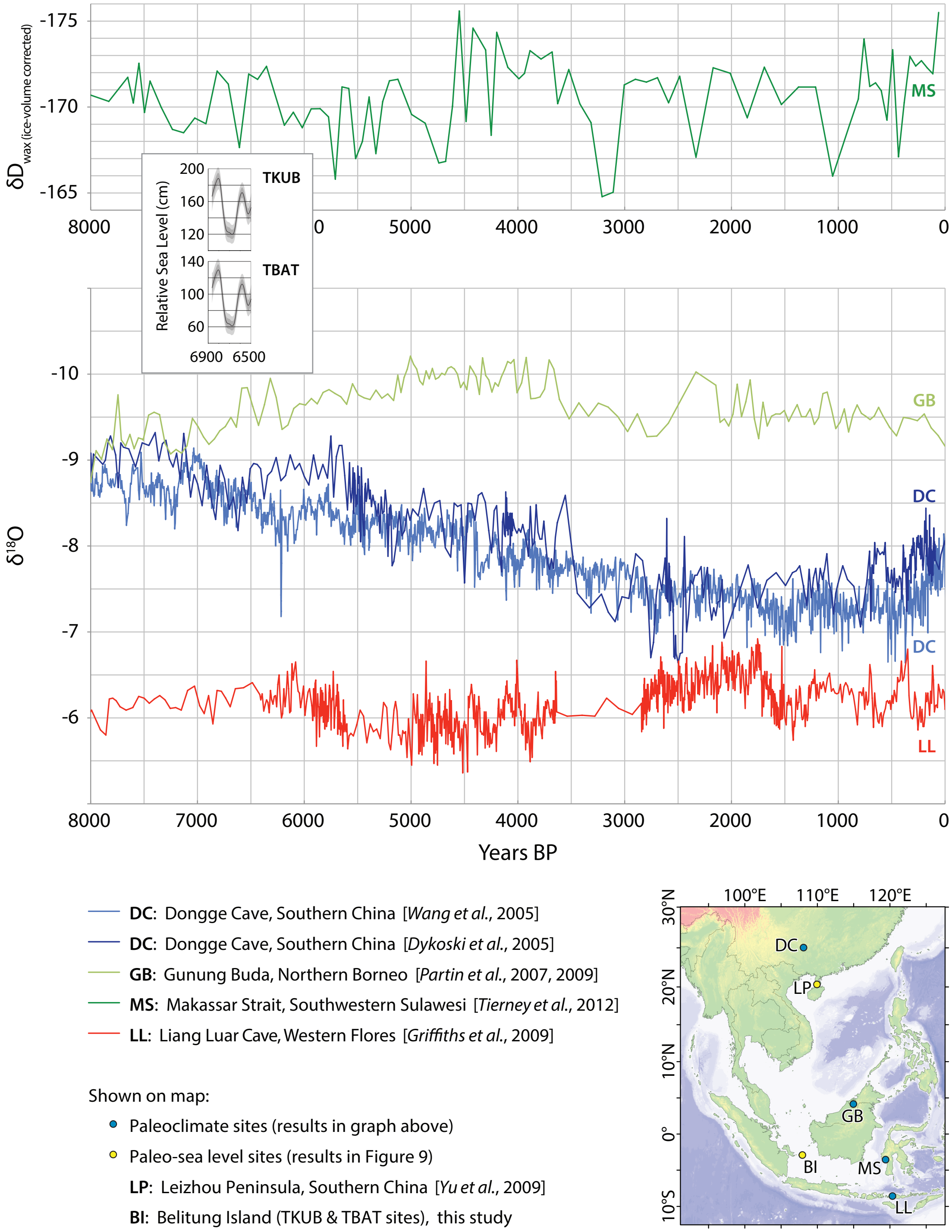

Supplementary Figure 5 | Comparison of RSL and paleoclimate proxy records.
The RSL time series from the Belitung sites (see detail on Supplementary Figures 8–9) aligned with paleoclimate proxy records from southern China[7,8], northern Borneo[9,10], southwestern Sulawesi[11], and western Flores[12]. $\delta^{18}O$ and $\delta D_{wax\ (ice\text{-}volume\ corrected)}$ are paleoclimate proxies defined in the respective papers. Map shows paleoclimate and paleo-sea level (microatoll) study sites as blue and yellow circles, respectively. Map created in ArcGIS.

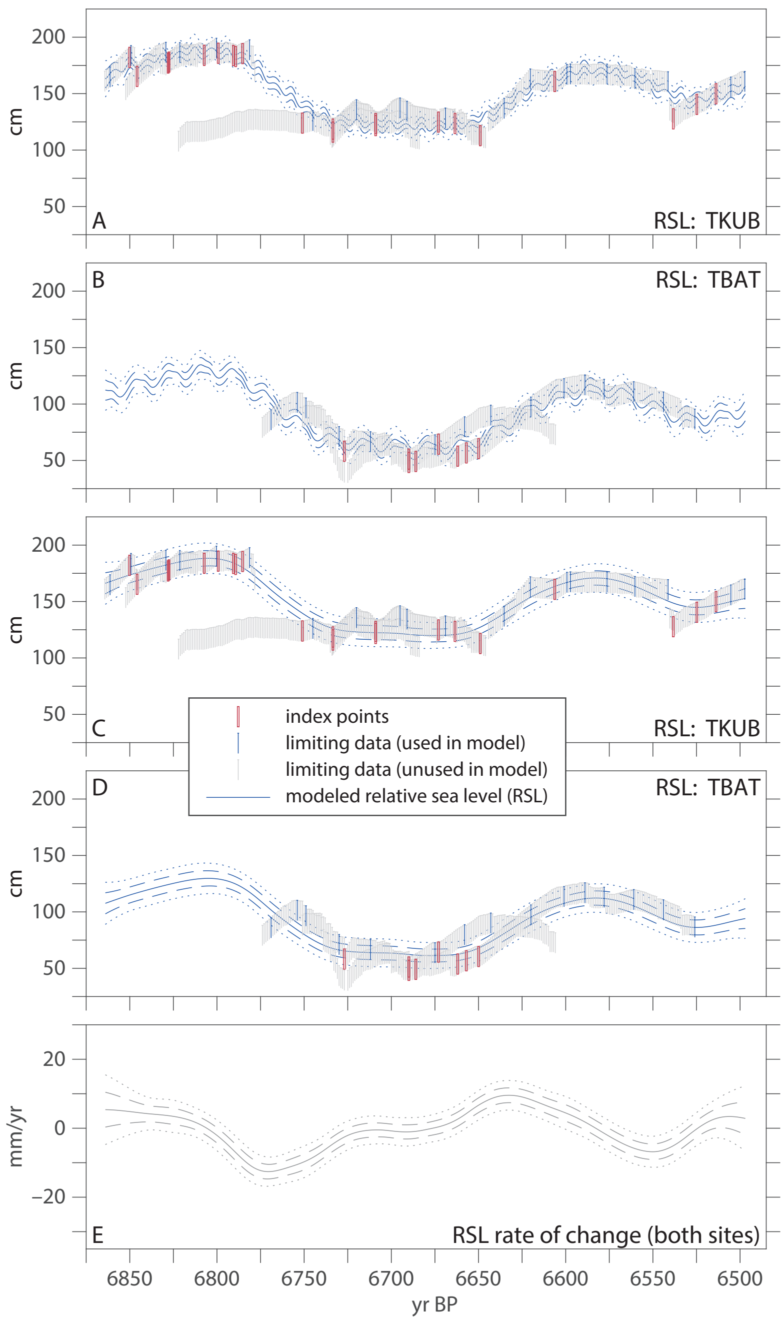

Supplementary Figure 6 | Modeled RSL based on a preferred subset of proxy data. Posterior sea-level curves based on an empirical hierarchical model that includes a non-linear term, common to both sites, in addition to a site-specific constant vertical shift and a periodic term, tuned to simulated coral growth over the 18.61-yr nodal tidal cycle at each site. The timing of the TBAT site time series is fixed to that determined using $\Delta R = +89$, but the three floating chronologies for TKUB are individually allowed to float relative to the TBAT time series, as described in the text. Here all index points and the highest limiting data point within each 18.61-yr bin are conditioned upon in the model. (**a**, **b**) The full model, for sites TKUB and TBAT, including the 18.61-yr periodic term for each site. (**c**, **d**) The model with periodic terms excluded, to show only secular trends in RSL, for TKUB and TBAT. The modeled curves in (**c**) and (**d**) are identical, except for a uniform shift in RSL between the two sites. (**e**) Rates of change, averaged over 20-yr running windows, determined from the model in (**c**) and (**d**). For the index points and limiting data, symbols show $\pm 1\sigma$ vertical error bars (± 9 cm) and $\pm 2\sigma$ relative timing errors (± 0.5 yr band-counting uncertainties). For the models, dashed and dotted curves depict $\pm 1\sigma$ and $\pm 2\sigma$ error envelopes, respectively.

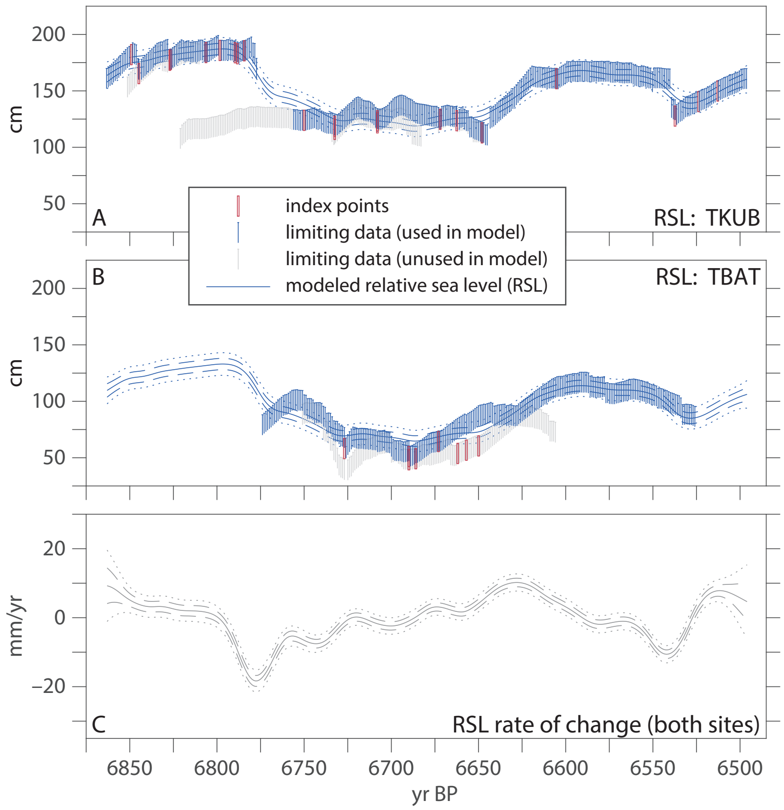

Supplementary Figure 7 | Modeled RSL based on a larger subset of proxy data.
Posterior sea-level curves based on an empirical hierarchical model that includes a non-linear term, common to both sites, in addition to a site-specific constant vertical shift and a periodic term, tuned to simulated coral growth over the 18.61-yr nodal tidal cycle at each site. The timing of the TBAT site time series is fixed to that determined using ΔR = +89, but the three floating chronologies for TKUB are individually allowed to float relative to the TBAT time series, as described in the text. Here all index points and the highest limiting data point available for each year (excluding the early part of TKUB-F16, before the coral had grown up to its highest level of survival, or HLS) are conditioned upon in the model. (**a**, **b**) The model with periodic terms excluded, to show only secular trends in RSL, for TKUB and TBAT. The modeled curves in (**a**) and (**b**) are identical, except for a uniform shift in RSL between the two sites. (**c**) Rates of change, averaged over 20-yr running windows, determined from the model in (**a**) and (**b**). For the index points and limiting data, symbols show ±1σ vertical error bars (±9 cm) and ±2σ relative timing errors (±0.5 yr band-counting uncertainties). For the models, dashed and dotted curves depict ±1σ and ±2σ error envelopes, respectively.

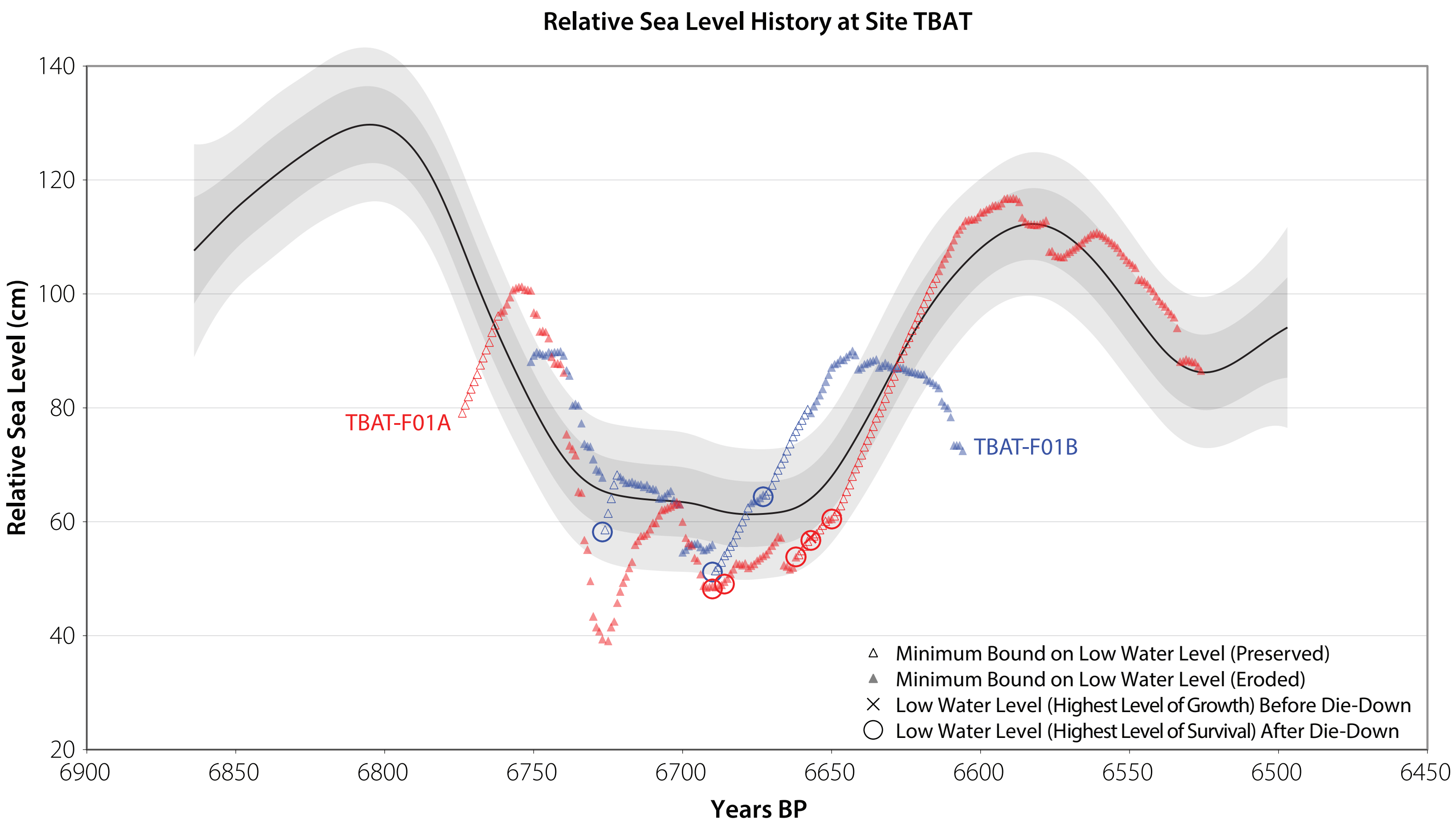

Supplementary Figure 8 | Mid-Holocene RSL proxy data and model for site TBAT.
Mid-Holocene RSL proxy time series determined from the TBAT-F01 microatoll at the TBAT site on southeastern Belitung. Data from each of two slabs are shown in a different color. The relative age uncertainty between two observations at this site is simply the annual band-counting uncertainty, commonly less than ±1 yr; however, unmodeled uncertainty in ΔR could affect absolute ages and would allow the entire curve to be shifted uniformly by up to ±85 yr. In particular, ΔR at TKUB may differ from ΔR at TBAT within the bounds of uncertainty. Overlain on the data is the posterior estimate of the common regional RSL signal, conditioned upon all index points (HLS data; open circles) and the highest minimum limiting data point (triangles) within each 18.61-yr bin. Vertical uncertainties about each data point are uniformly ±9 cm (1σ) but are not shown here for clarity. For the models, dark and light shading depict ±1σ and ±2σ error envelopes, respectively.

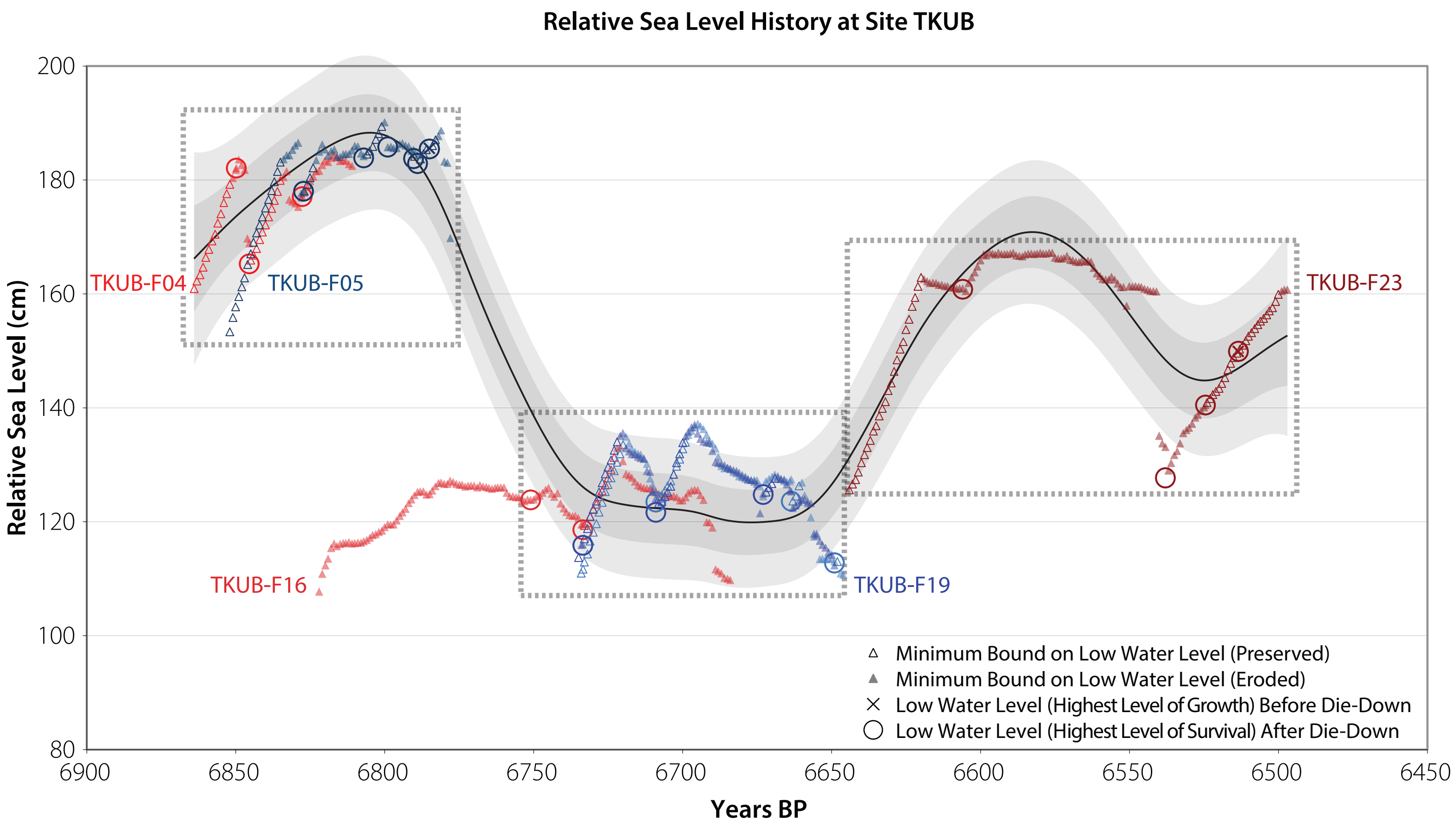

Supplementary Figure 9 | Mid-Holocene RSL proxy data and model for site TKUB.
Mid-Holocene RSL proxy time series determined from coral microatolls at the TKUB site on northwestern Belitung, as in Figure 8, but with the timing of each discrete floating chronology (indicated by a dotted box) optimized by our model relative to the timing of the TBAT time series (Figure 6). Colors correspond to data from different corals. ΔR did not vary over the lifetime of corals at this site, and therefore uncertainty in ΔR can be ignored when calculating the relative (differential) age of these corals; however, unmodeled uncertainty in ΔR could affect absolute ages and would allow the entire curve to be shifted uniformly by up to ±85 yr. In particular, ΔR at TKUB may differ from ΔR at TBAT within the bounds of uncertainty. Overlain on the data is the posterior estimate of the common regional RSL signal, conditioned upon all index points (HLS data; open circles) and the highest minimum limiting data point (triangles) within each 18.61-yr bin. Vertical uncertainties about each data point are uniformly ±9 cm (1σ) but are not shown here for clarity. For the models, dark and light shading depict ±1σ and ±2σ error envelopes, respectively.

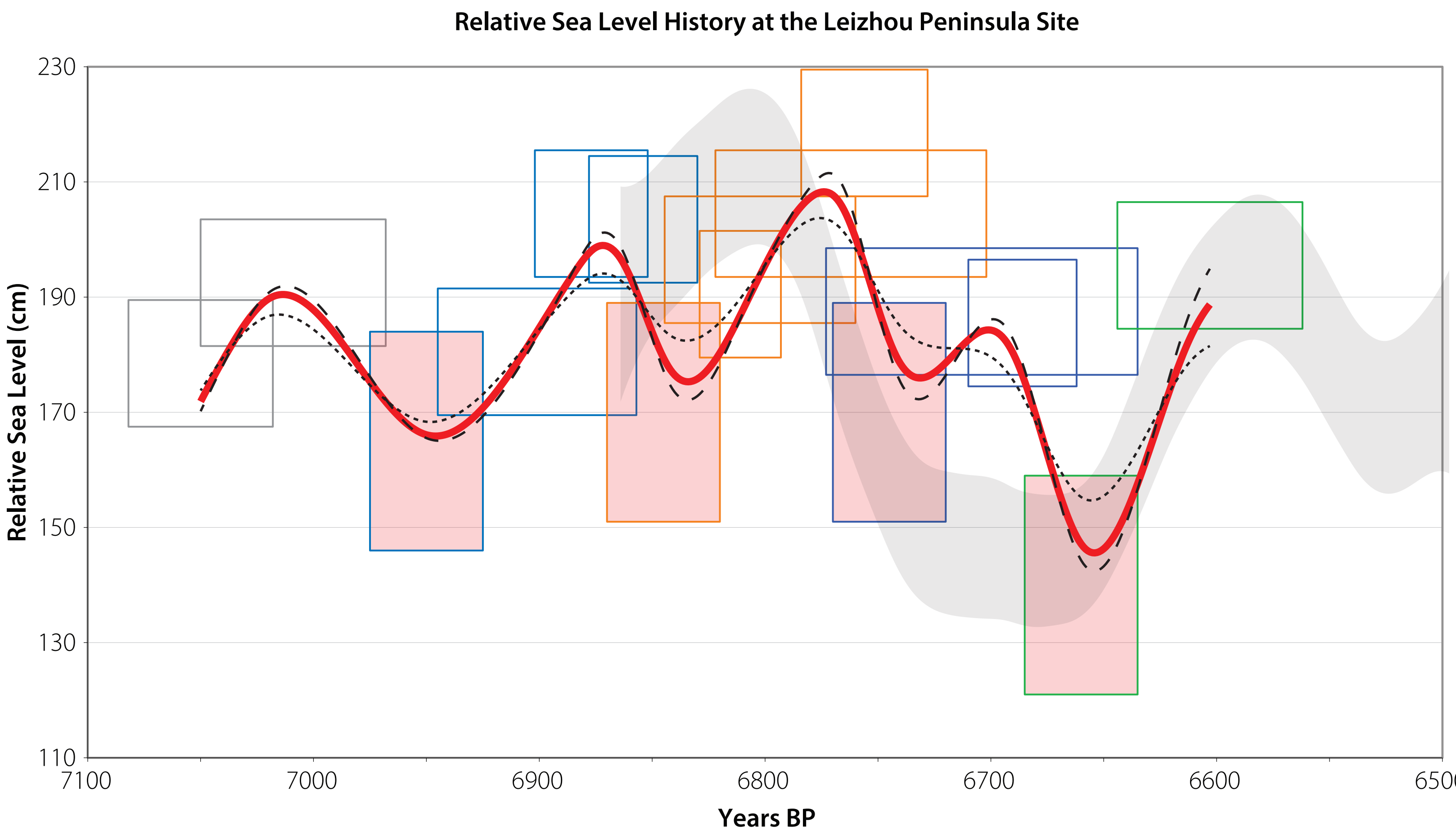

Supplementary Figure 10 | Mid-Holocene RSL proxy data and models for the Leizhou Peninsula site. Reinterpreted RSL history at the Leizhou Peninsula site of Yu et al.[13]. The boxes with shaded red fill represent the four diedowns reflected in the microatoll morphology at the site. Dated samples, each tied to a surveyed elevation, are shown by open boxes, with 1σ vertical errors and 2σ temporal errors. All boxes are color-coded (for ease of visualization) to correspond to one of the sequential sea-level drops required by microatoll diedowns at the site, and the subsequent sea-level rise. Yu et al.[13] did not consider the diedowns in their interpretation, nor did they consider the detailed morphology of the individual microatolls, but observations presented in their paper allow us to reconstruct the history as shown here. The solid red curve is our preferred model, with amplitude hyperparameters scaled by 2, as discussed in the Methods; dotted and dashed curves are the unscaled model, and the model with amplitude hyperparameters scaled by 3. Overlain on the plot is the Belitung RSL curve from Supplementary Figures 8–9 (2σ error envelope in gray); the Belitung curve is shifted vertically to optimize the fit, but there has been no temporal shift. A different choice of ΔR for the TBAT site, which pins the timing of the Belitung chronology, would allow for a temporal shift of the Belitung curve and could improve the agreement between the sites further.

Supplementary Table 1 | Locations of slabbed coral microatolls.

Microatoll name	Latitude (°N)	Longitude (°E)
TBAT-F01	-3.23278	108.08151
TKUB-F04	-2.69603	107.61795
TKUB-F05	-2.69605	107.61789
TKUB-F16	-2.69446	107.61704
TKUB-F19	-2.69785	107.61622
TKUB-F23	-2.68197	107.62744

Supplementary Table 2 | Radiocarbon dates and OxCal analysis.

Microatoll / Sample Name	Radiocarbon Age[1] (rcy BP)		Unmodeled Age[2] (yr BP)		No. of Younger Annual Bands[3]	Unmodeled Age of Outer Band[4] (yr BP)			Modeled Age[2] (yr BP)	
	median	σ	median	σ		median	oldest (1σ)	youngest (1σ)	median	σ
TBAT-F01A-J1	6431	20	6806	40	247	6559	6599	6519	6773	13
TBAT-F01A-I1	6355	20	6714	37	242	6472	6509	6435	6768	13
TBAT-F01B-Q1	6391	21	6757	41	232	6525	6566	6484	6758	13
TBAT-F01B-O1	6396	20	6763	41	215	6548	6589	6507	6741	13
TBAT-F01B-N1	6369	20	6730	39	174	6556	6595	6517	6700	13
TBAT-F01A-E1	6298	20	6655	37	153	6502	6539	6465	6679	13
TBAT-F01B-K1	6281	20	6636	39	101	6535	6574	6496	6627	13
TBAT-F01A-C1	6228	20	6573	40	68	6505	6545	6465	6594	13
**TBAT-F01 outer band**									**6526**	**13**
TKUB-F04-CB	6435	24	6811	43	48	6763	6806	6720	6785	33
TKUB-F04-CA	6358	32	6719	47	11	6708	6755	6661	6748	33
**TKUB-F04 outer band**									**6737**	**33**
TKUB-F05-CC-D2	6392	27	6759	46	74	6685	6731	6639	6757	21
TKUB-F05-CC-D1	6419	27	6792	46	73	6719	6765	6673	6756	21
TKUB-F05-CC-A2	6361	27	6721	43	71	6650	6693	6607	6754	21
TKUB-F05-CC-A1	6389	28	6755	46	70	6685	6731	6639	6753	21
**TKUB-F05 outer band**									**6683**	**21**
TKUB-F16-CB	6433	28	6808	47	128	6680	6727	6633	6791	29
TKUB-F16-CA	6328	22	6686	36	33	6653	6689	6617	6696	29
**TKUB-F16 outer band**									**6663**	**29**
TKUB-F19-CB	6290	22	6646	40	89	6557	6597	6517	6648	29
TKUB-F19-CA	6233	28	6577	46	20	6557	6603	6511	6579	29
**TKUB-F19 outer band**									**6559**	**29**
TKUB-F23-CB	6270	21	6622	40	128	6494	6534	6454	6630	29
TKUB-F23-CA	6226	22	6571	42	63	6508	6550	6466	6565	29
**TKUB-F23 outer band**									**6502**	**29**

[1] Conventional radiocarbon ages are from Rafter Radiocarbon Laboratory, GNS Science, and are reported as defined by *Stuiver and Polach* [1977].
The reported errors comprise statistical errors in sample and standard determinations, combined in quadrature with a system error component based on the analysis of an ongoing series of measurements on an oxalic acid standard. Further analysis details are available on request.

[2] The radiocarbon dates were modeled using the OxCal calibration program [*Bronk Ramsey*, 2008].
We applied the Marine13 radiocarbon age calibration curve [*Reimer et al.*, 2013], assuming ΔR = +89 yr.
The 'unmodeled age' is corrected for ΔR but is based only on the individual sample indicated; it is independent of all other samples from the slab.
The 'modeled age' is corrected for ΔR and accounts for all samples on the slab and their respective separations.

[3] This is the number of preserved annual growth bands on the slab that are younger than the sample indicated.

[4] This is the unmodeled age of the individual sample, minus the number of younger annual growth bands on the slab, plus or minus 1σ.
This is an estimate of the age of the outer preserved band of the slab, based only on the sample indicated, and independent of all other samples from the slab.

Supplementary Table 2 | Radiocarbon dates and OxCal analysis.

Eight samples were dated from the TBAT-F01 coral, and at least two samples were dated from each coral at the TKUB site. Conventional radiocarbon ages[14] were modeled using the OxCal calibration program[15]. We applied the Marine13 radiocarbon age calibration curve[16], assuming the marine reservoir correction ΔR ≈ +89 yr, based on a ΔR value established from an early 20th-century sample from southwestern Borneo[17,18]. Although considerable uncertainty exists in any ΔR value and its extrapolation spatially (and to mid-Holocene samples), we can establish that, whatever ΔR was at our sites at the time, it did not vary in a statistically significant manner over the lifetimes of our mid-Holocene corals. For each slab, the 2–8 ages listed under "unmodeled age of outer band" are independent estimates of the age of the outer preserved band of that slab based on individual samples. For the TBAT slab, seven of the eight ages agree at 1σ, and all agree at 2σ, with the median modeled age for the outer band. For all of the TKUB slabs, all ages agree at 1σ with the median modeled age for the outer band. This is better than expected based on the laboratory errors and the calibration curve: as a rule, only 68% of data should agree at 1σ, and 95% should agree at 2σ. Although ΔR at TKUB may differ from ΔR at TBAT, the remarkable agreement amongst the unmodeled dates from each slab precludes significant temporal variation in ΔR over the lifetime of each coral; if the marine reservoir correction varied by more than a few decades, we would not expect such consistency amongst the radiocarbon dates.

Supplementary Table 3 | Optimized model parameters and hyperparameters.

		Parameters				Hyperparameters[1]					
		c_j	Δ_0	Δ_1	Δ_2	σ_g	τ	σ_p	ν_p	σ_c	σ_w
Limiting data (LD) included in model (in addition to all index points):	cf. Supp. Fig. #	Constant Site-Specific Offset (mm)	Age shift: entire TKUB record (yr)	Age shift: TKUB-F04 & TKUB-F05 (yr)	Age shift: TKUB-F23 (yr)	Common Signal St. Dev. Amplitude (mm)	Timescale of Variability (yr)	Periodic Signal St. Dev. Amplitude (mm)	Smoothness Parameter	Site Offset St. Dev. Amplitude (mm)	White Noise St. Dev. Amplitude (mm)
Highest LD per nodal cycle	6	590.0	54	20	1	878.1	182.6	66.4	0.98	69.9	< 0.1
Highest LD per year	7	544.5	53	20	1	861.9	127.0	66.4	0.98	63.2	< 0.1

[1] σ_p and ν_p were optimized based on coral growth models (Figure 2) and held constant during optimization of the other hyperparameters.

Supplementary Table 4 | Modeled rates of RSL change.

a) Rates averaged over 20-yr running windows

Limiting data (LD) included in model (in addition to all index points):	cf. Supp. Fig. #	Maximum (Peak) Rate of RSL RISE			Maximum (Peak) Rate of RSL FALL		
		**Rate** (mm/yr)	**Std. Dev.** (mm/yr)	**Timing** (yr BP)	**Rate** (mm/yr)	**Std. Dev.** (mm/yr)	**Timing** (yr BP)
Highest LD per nodal cycle	6	+ 9.6	± 2.1	6642-6622	– 12.6	± 2.1	6780-6760
Highest LD per year	7	+ 10.2	± 1.2	6639-6619	– 18.3	± 1.6	6788-6768

b) Rates averaged over 10-yr running windows

Limiting data (LD) included in model (in addition to all index points):	cf. Supp. Fig. #	Maximum (Peak) Rate of RSL RISE			Maximum (Peak) Rate of RSL FALL		
		**Rate** (mm/yr)	**Std. Dev.** (mm/yr)	**Timing** (yr BP)	**Rate** (mm/yr)	**Std. Dev.** (mm/yr)	**Timing** (yr BP)
Highest LD per nodal cycle	—	+ 9.8	± 2.7	6636-6626	– 13.1	± 2.7	6777-6767
Highest LD per year	—	+ 11.1	± 2.1	6631-6621	– 23.2	± 2.5	6783-6773

Supplementary Table 5 | Rheological parameters used in GIA models and differential RSL change across Belitung.

Earth model name	96p28	9611	96p38	96p250	96p130	96p510
Lithosphere thickness (km)	96	96	96	96	96	96
Upper mantle viscosity (Pa·s)	0.2×10^{21}	1.0×10^{21}	0.3×10^{21}	0.2×10^{21}	0.1×10^{21}	0.5×10^{21}
Lower mantle viscosity (Pa·s)	8.0×10^{21}	1.0×10^{21}	8.0×10^{21}	50×10^{21}	30×10^{21}	10×10^{21}
Chi squared misfit (to Malay–Thai data)	15.61	16.99	18.75	20.72	22.05	25.47
Difference in RSL at 7 kyr BP between TKUB and TBAT (m)	0.23	0.40	0.31	0.23	0.12	0.39

A selection of earth model parameters used in the χ^2 analysis of Bradley et al.[1], and the corresponding predictions of differential RSL change across Belitung. For all models, features formed 7 kyr BP at RSL would be higher today at the northwestern site (TKUB) than at the southeastern site (TBAT).

Supplementary Table 6 | Rheological parameters used in postseismic viscoelastic models.

**High-viscosity model [*Pollitz et al.*, 2006, 2008]**			
Layer	Upper depth (km)	Lower depth (km)	Viscosity (Pa·s)
Crust (elastic)	0	62	1×10^{29} (effectively elastic)
Asthenosphere (Burgers body)	62	220	5×10^{17} (transient)<br>1×10^{19} (Maxwell)
Upper mantle (Maxwell)	220	670	1×10^{20}
Lower mantle (Maxwell)	670	2891	1×10^{21}

**Low-viscosity model [*Panet et al.*, 2010]**			
Layer	Upper depth (km)	Lower depth (km)	Viscosity (Pa·s)
Crust (elastic)	0	60	1×10^{29} (effectively elastic)
Asthenosphere (Burgers body)	60	220	4×10^{17} (transient)<br>8×10^{18} (Maxwell)
Upper mantle (Maxwell)	220	670	8×10^{18}
Lower mantle (Maxwell)	670	2891	8×10^{20}

Supplementary Table 7 | Original and reinterpreted data from the Leizhou Peninsula site.

Grouping[1]	Microatoll[2]	Sample[2]	U-Th Age[2] ± 2σ (yr BP)	Modeled Age[3] ± 2σ (yr BP)	Elevation[2,4] ± 1σ (cm)
1	5	FPO-41	7050 ± 32	7038 ± 32	178.5 ± 11
1	3	FPO-31	7009 ± 41	7019 ± 36	192.5 ± 11
2	estimated diedown		6950 ± 25	6950 ± 24	165.0 ± 19
3	5	FPO-40	6901 ± 44	6884 ± 36	180.5 ± 11
3	4	FPO-37	6877 ± 25	6875 ± 22	204.5 ± 11
3	3	FPO-29	6854 ± 24	6864 ± 20	203.5 ± 11
4	estimated diedown		6845 ± 25	6842 ± 20	170.0 ± 19
5	2	FPO-36	6811 ± 18	6808 ± 18	190.5 ± 11
5	3	FPO-26/30	6802 ± 21	6798 ± 32	196.5 ± 11
5	4	FPO-38	6762 ± 60	6779 ± 38	204.5 ± 11
5	2	FPO-35	6756 ± 28	6767 ± 24	218.5 ± 11
6	estimated diedown		6745 ± 25	6741 ± 22	170.0 ± 19
7	1	FPO-22	6704 ± 69	6695 ± 34	187.5 ± 11
7	3	FPO-27	6686 ± 24	6689 ± 22	185.5 ± 11
8	estimated diedown		6660 ± 25	6659 ± 22	140.0 ± 19
9	1	FPO-23	6603 ± 41	6612 ± 40	195.5 ± 11

[1] This is a sequential grouping of the data, based on coral morphology.
Samples within a group are unordered, but the groups themselves are ordered.
This ordering was not considered by *Yu et al.* [2009] in their interpretations, but it is apparent in their data.

[2] For groupings 1, 3, 5, 7, 9: these values are as reported by *Yu et al.* [2009].
For groupings 2, 4, 6, 8: these values are inferred by us based on the observations of *Yu et al.* [2009].
Sample FPO-26/30 is a weighted average of samples FPO-26 and FPO-30, as reported by *Yu et al.* [2009].

[3] The ages were modeled using OxCal [*Bronk Ramsey*, 2008].
Each grouping was classified as a Phase() and was separated from adjacent groupings by a Boundary().

[4] The vertical errors here are increased from those reported by *Yu et al.* [2009], as discussed in the text.

Supplementary Table 8 | Model cross validation.

		95% prediction interval			Errors (mm)		
	Observations	Below	Within	Above	Mean Error	Median Error	Median Absolute Error
Actual	Count (out of 64)	2	59	3	-1.1	7.5	64.7
	Percentage	3.1%	92.2%	4.7%			
Expected	Count (out of 64)	1.6	60.8	1.6	0	0	
	Percentage	2.5%	95.0%	2.5%			

Supplementary Note 1 | Possible correlations with regional hydroclimate.

We explore possible correlations between the fluctuations observed in the mid-Holocene RSL proxy data and published regional hydroclimate histories. Paleoclimate proxy records from southern China[7,8], northern Borneo[9,10], southwestern Sulawesi[11], and western Flores[12] are shown in Supplementary Figure 5. These records differ markedly from one another, both in long-term trends and in detail, suggesting substantial hydroclimate heterogeneity across the region. Changes in the respective records' isotope data likely reflect changes in a combination of factors, including the strength of the Asian (and East Asian) monsoon, the position of the Inter-Tropical Convergence Zone (ITCZ), Indo-Pacific Walker circulation, and orbital forcing[11].

We note that the paleoclimate proxy records from the two sites closest to Belitung—in Borneo[9,10] and in Sulawesi[11]—show large fluctuations at approximately the same time as the sea-level fluctuations we observe on Belitung. Given the moderate uncertainties in the timing of the Belitung fluctuations (due to uncertainty in ΔR) and of the Borneo record (as acknowledged by Partin et al.[9,10]), the Belitung sea-level fluctuations may correlate with the large paleoclimate fluctuations in either Borneo or Sulawesi. That said, we note that the Borneo and Sulawesi fluctuations around 6.5 ka are not the largest in either record, and other large fluctuations occur in each record. If the Borneo and/or Sulawesi paleoclimate fluctuations are indeed related to the RSL fluctuations at Belitung, we might expect similar sea-level fluctuations at Belitung at various times since 6.5 ka. This would be a testable hypothesis, if sufficiently high-resolution RSL proxy records could be obtained from Belitung spanning several of those younger periods.

Supplementary References

1 Bradley, S. L., Milne, G. A., Horton, B. P. & Zong, Y. Modelling sea level data from China and Malay–Thailand to estimate Holocene ice-volume equivalent sea level change. *Quat. Sci. Rev.* **137**, 54-68, doi:10.1016/j.quascirev.2016.02.002 (2016).

2 Zong, Y. Mid-Holocene sea-level highstand along the southeast coast of China. *Quat. Int.* **117**, 55-67, doi:10.1016/S1040-6182(03)00116-2 (2004).

3 Horton, B. P. *et al.* Holocene sea levels and palaeoenvironments, Malay–Thai Peninsula, Southeast Asia. *Holocene* **15**, 1199-1213, doi:10.1191/0959683605hl891rp (2005).

4 Pollitz, F. F., Bürgmann, R. & Banerjee, P. Post-seismic relaxation following the great 2004 Sumatra–Andaman earthquake on a compressible self-gravitating Earth. *Geophys. J. Int.* **167**, 397-420, doi:10.1111/j.1365-246X.2006.03018.x (2006).

5 Pollitz, F., Banerjee, P., Grijalva, K., Nagarajan, B. & Bürgmann, R. Effect of 3-D viscoelastic structure on post-seismic relaxation from the 2004 $M = 9.2$ Sumatra earthquake. *Geophys. J. Int.* **173**, 189-204, doi:10.1111/j.1365-246X.2007.03666.x (2008).

6 Panet, I. *et al.* Upper mantle rheology from GRACE and GPS postseismic deformation after the 2004 Sumatra–Andaman earthquake. *Geochem. Geophys. Geosyst.* **11**, Q06008, doi:10.1029/2009GC002905 (2010).

7 Wang, Y. *et al.* The Holocene Asian monsoon: links to solar changes and North Atlantic climate. *Science* **308**, 854-857, doi:10.1126/science.1106296 (2005).

8 Dykoski, C. A. *et al.* A high-resolution, absolute-dated Holocene and deglacial Asian monsoon record from Dongge Cave, China. *Earth Planet. Sci. Lett.* **233**, 71-86, doi:10.1016/j.epsl.2005.01.036 (2005).

9 Partin, J. W., Cobb, K. M., Adkins, J. F., Clark, B. & Fernandez, D. P. Millennial-scale trends in west Pacific warm pool hydrology since the Last Glacial Maximum. *Nature* **449**, 452-455, doi:10.1038/nature06164 (2007).

10 Partin, J. W., Cobb, K. M., Adkins, J. F., Clark, B. & Fernandez, D. P. Corrigendum: Millennial-scale trends in west Pacific warm pool hydrology since the Last Glacial Maximum. *Nature* **459**, 736, doi:10.1038/nature08125 (2009).

11 Tierney, J. E. *et al.* The influence of Indian Ocean atmospheric circulation on Warm Pool hydroclimate during the Holocene epoch. *J. Geophys. Res.* **117**, D19108, doi:10.1029/2012JD018060 (2012).

12 Griffiths, M. L. *et al.* Increasing Australian–Indonesian monsoon rainfall linked to early Holocene sea-level rise. *Nat. Geosci.* **2**, 636-639, doi:10.1038/ngeo605 (2009).

13 Yu, K.-F., Zhao, J.-X., Done, T. & Chen, T.-G. Microatoll record for large century-scale sea-level fluctuations in the mid-Holocene. *Quat. Res.* **71**, 354-360, doi:10.1016/j.yqres.2009.02.003 (2009).

14 Stuiver, M. & Polach, H. A. Discussion: reporting of C-14 data. *Radiocarbon* **19**, 355-363 (1977).

15 Bronk Ramsey, C. Deposition models for chronological records. *Quat. Sci. Rev.* **27**, 42-60, doi:10.1016/j.quascirev.2007.01.019 (2008).

16 Reimer, P. J. *et al.* IntCal13 and Marine13 radiocarbon age calibration curves 0–50,000 years cal BP. *Radiocarbon* **55**, 1869-1887, doi:10.2458/azu_js_rc.55.16947 (2013).

17 Southon, J., Kashgarian, M., Fontugne, M., Metivier, B. & Yim, W. W.-S. Marine reservoir corrections for the Indian Ocean and Southeast Asia. *Radiocarbon* **44**, 167-180 (2002).

18 ^{14}Chrono Centre for Climate, the Environment, and Chronology. ^{14}CHRONO Marine Reservoir Database, http://calib.org/marine/ (2016).
